# Impact of anti-CD25 monoclonal antibody on dendritic cell-tumor fusion vaccine efficacy in a murine melanoma model

**DOI:** 10.1186/1479-5876-11-148

**Published:** 2013-06-17

**Authors:** Chunrui Tan, Varun Reddy, Jens Dannull, Enyu Ding, Smita K Nair, Douglas S Tyler, Scott K Pruitt, Walter T Lee

**Affiliations:** 1 Division of Otolaryngology, Duke University Medical Center, Durham, USA; 2 Duke University School of Medicine, Durham, USA; 3 Department of Surgery, Duke University Medical Center, Durham, USA; 4 Department of Surgical Sciences, Duke University Medical Center, Durham, USA; 5 Department of Surgery, Durham VA Medical Center, Durham, USA

**Keywords:** Regulatory T cell, Fusion, PC61, Dendritic cell, Tumor, Immunotherapy

## Abstract

**Background:**

A promising cancer vaccine involves the fusion of tumor cells with dendritic cells (DCs). As such, a broad spectrum of both known and unidentified tumor antigens is presented to the immune system in the context of the potent immunostimulatory capacity of DCs. Murine studies have demonstrated the efficacy of fusion immunotherapy. However the clinical impact of DC/tumor fusion vaccines has been limited, suggesting that the immunosuppresive milieu found in patients with malignancies may blunt the efficacy of cancer vaccination. Thus, novel strategies to enhance fusion vaccine efficacy are needed. Regulatory T cells (Tregs) are known to suppress anti-tumor immunity, and depletion or functional inactivation of these cells improves immunotherapy in both animal models and clinical trials. In this study, we sought to investigate whether functional inactivation of CD4+CD25+FoxP3+ Treg with anti-CD25 monoclonal antibody (mAb) PC61 prior to DC/tumor vaccination would significantly improve immunotherapy in the murine B16 melanoma model.

**Methods:**

Treg blockade was achieved with systemic PC61 administration. This blockage was done in conjunction with DC/tumor fusion vaccine administration to treat established melanoma pulmonary metastases. Enumeration of these metastases was performed and compared between experimental groups using Wilcoxon Rank Sum Test. IFN-gamma ELISPOT assay was performed on splenocytes from treated mice.

**Results:**

We demonstrate that treatment of mice with established disease using mAb PC61 and DC/tumor fusion significantly reduced counts of pulmonary metastases compared to treatment with PC61 alone (p=0.002) or treatment with control antibody plus fusion vaccine (p=0.0397). Furthermore, IFN-gamma ELISPOT analyses reveal that the increase in cancer immunity was mediated by anti-tumor specific CD4+ T-helper cells, without concomitant induction of CD8+ cytotoxic T cells. Lastly, our data provide proof of principle that combination treatment with mAb PC61 and systemic IL-12 can lower the dose of IL-12 necessary to obtain maximal therapeutic efficacy.

**Conclusions:**

To our knowledge, this is the first report investigating the effects of anti-CD25 mAb administration on DC/tumor-fusion vaccine efficacy in a murine melanoma model, and our results may aide the design of future clinical trials with enhanced therapeutic impact.

## Introduction

Melanoma kills over 8,000 people in the US each year, and its incidence is increasing faster than that of any other malignancy, with over 65,000 diagnosed cases last year (NCI cancer statistics). The mainstay of treatment remains surgical excision of the primary skin lesion, with regional lymph node dissection to remove nodal metastases. For subjects with isolated nodal metastases at presentation, the 5 year survival rate is less than 50%, and standard treatments in both the adjuvant and inoperable metastatic disease settings show little to no survival benefit.

In contrast, immunotherapy with DC-tumor cell fusion vaccines represents a particularly promising approach for the treatment of metastatic melanoma. Melanoma cells express a wide spectrum of known as well as patient-specific tumor-associated antigens and DCs are the most potent antigen-presenting cells attributable to their abundant expression of major histocompatibility complex (MHC) class I and II molecules as well as co-stimulatory and adhesion molecules which provide signals 1 and 2 for stimulation of naïve T cells [1]. However, the induction of primary T-cell responses against tumor-associated antigens *in vivo* is critically dependent upon co-administration of an adjuvant that can provide a 3^rd^ signal to prevent T-cell tolerance or anergy and hence to induce T-cell effector function and memory [2]. 3^rd^ signals include systemic IL-12 [3], agonistic antiOX40- [4], anti4-1BB- [5] or antagonistic PD-1 [6] monoclonal antibodies (mAbs), as well as DC1-polarizing Toll-like receptor agonists such as poly(I:C) and CpG. [7] The success of DC/fusion-based cancer vaccination in animal models has prompted the initiation of several clinical trials, but despite potent induction of anti-tumor T-cell immunity, only modest clinical responses were observed in a minority of patients [8]. Thus, it is evident that additional strategies are needed to improve fusion vaccine efficacy.

A major obstacle to the development of any immunotherapeutic approach is the control of immune-balance. Regulatory T cells (Tregs) are responsible for maintaining tolerance to self-antigens, and immune homeostasis by regulating the activation of non-regulatory T cells [9]. Tregs exert their effects through TGF-β [10], IL-10 [11], CTLA-4 (cytotoxic T-lymphocyte antigen 4) [12], or through sequestration of IL-2 via expression of CD25 [13], the α-subunit of the high-affinity IL-2 receptor, and are defined by their expression of the transcription factor forkhead box transcription factor 3 (FoxP3) [14]. Recently, several methods for the depletion or functional inactivation of Tregs have been developed as part of a multi-pronged approach to the immunotherapy of melanoma patients. Treatment with low dose cyclophosphamide resulted in a reduction of Treg frequencies [15], however Treg elimination was also associated with a concomitant reduction of CD8^+^ T cells and a lack of tumor antigen priming [16]. Administration of CD25-targeted immunotoxins, designed to have a direct cytocidal action on cells which express the high-affinity IL-2 receptor, only leads to a modest and transient reduction in Treg numbers and has achieved variable results in improving immunotherapy [17,18]. The fully human mAb Ipilimumab was approved in 2011 by the FDA for clinical use against melanoma. Ipilumumab exerts its therapeutic effects through direct enhancement of CD8^+^ T-cell function and simultaneous inhibition of Treg function through blockade of CTLA-4 on both cell types [19], which makes it hard to delineate its effects on a DC vaccine that aims to induce primary immune responses against tumor-associated antigens. Lastly, the anti-mouse CD25 mAb PC61, as well as the anti-human CD25 mAbs Basiliximab and Daclizumab, demonstrated a potent deactivation of Treg suppression that was mediated through moderate reduction of Treg numbers [20-23] and functional inhibition through blockade of IL-2 signaling [24]. Importantly, treatment with anti-CD25 mAbs did not abrogate tumor antigen-specific immunity elicited by DC-based vaccines, despite the fact that activated effector T cells transiently express high levels of CD25. We therefore chose to use mAb PC61 in our vaccination studies.

In this report, we demonstrate that administration of mAb PC61 was an effective means to functionally inactivate Tregs in a murine melanoma model. In addition, we provide evidence that inactivation of Tregs enhances the potency of vaccination with DC/tumor cell fusions in a synergistic fashion, and that the enhancement of anti-tumor activity was primarily mediated by CD4^+^ T-effector cells. Furthermore, our data provide proof of principle that combination treatment with mAb PC61 and systemic IL-12 can lower the dose of cytokine necessary to obtain maximal therapeutic efficacy and may therefore represent a viable strategy to reduce IL-12 mediated toxicity in a clinical setting.

## Materials and methods

### Animals

This work was approved by the Duke University School of Medicine and Durham VA Medical Center IACUC. Female C57BL/6 mice were purchased from Charles River Laboratories (Raleigh, NC). Animals were maintained in a specific pathogen-free environment and used for experiments at age 8 to 12 weeks.

### Tumor cells

D5lacZ is a derivative of the B16 melanoma cell line which stably expresses the lacZ gene product β-galactosidase. These cells are cultured in complete medium (CM, RPMI 1640, 10% fetal bovine serum, 2 mM L-glutamine, 0.1 mM non-essential amino acids, 1 mM sodium pyruvate, 100 U/ml penicillin, 100 mg/ml streptomycin, 0.5 μg/ml fungizone, 50 μg/ml gentamicin and 5 × 10^-5^ M 2-mercaptoethanol) (Invitrogen, Grand Island, NY).

### Preparation of antibody

Hybridoma cells expressing anti-CD25 PC61 or Y13 (anti-HaRas) control mAbs were cultured in Hybridoma Serum-Free Media (Invitrogen) with 1% Penicillin/Streptomycin (Invitrogen), at the Duke University Cell Culture Facility. Antibodies were purified by ammonium sulfate precipitation of supernatants.

### Regulatory T-cell inactivation

The efficacy of the anti-CD25 PC61 mAb to deplete Treg was tested in both naïve and tumor bearing mice. 250 μg/0.5 ml Hank’s Balanced Salt Solution (HBSS) of PC61 or Y13 mAb was injected intraperitoneally into naive C3H/HeN mice. One mouse from each group was sacrificed at 7, 14, 21, 28, and 42 days after antibody injection. Spleens were harvested and enriched for T cells using the EasySep T-cell isolation kit (Stemcell Technologies, Vancouver, BC, Canada). T cells were stained for fluorescence-activated cell sorting (FACS) analysis with FITC-CD4, APC-CD25, and PE-FoxP3, respectively (eBioscience, San Diego, CA). Frequencies of CD4^+^CD25^+^FoxP3^+^ populations in both groups were compared to determine Treg inactivation. In order to confirm that the PC61 mAb was also effective in tumor-bearing mice, a dose of 0.25×10^6^ D5lacZ cells was injected via tail vein into C57BL/6 mice on Day 0. On Day 2, 250 μg/0.5 ml of PC61 or Y13 mAb were administered intraperitoneally. Mice were sacrificed at 3, 10, 20 days after tumor inoculation and Treg inactivation was determined by FACS.

### Dendritic cell preparation

DCs were generated from the femoral and tibial bone marrow cells of female C57BL/6 mice, After depletion of B and T cells using monoclonal antibody-coated magnetic beads (Invitrogen Dynal AS, Oslo, Norway), cells were cultured at 0.5×10^6^ cells/ml in CM supplemented with 10 ng/ml granulocyte macrophage colony-stimulating factor (GM-CSF) and 10 ng/ml IL-4 (Peprotech, Rocky Hill, NJ). On day 6, DCs were harvested and cultured in fresh CM+GM-CSF+IL-4 at 1×10^6^ cells/ml. One day 7, 25 ng/ml of lipopolysaccharide (Sigma-Aldrich, Saint Louis, MO) was added to the cultured cells to stimulate DC maturation. After 24 hours, DCs were collected for electrofusion.

### Electrofusion of dendritic cell-tumor hybrids

Irradiated D5lacZ cells (100 Gy) were stained green with intracellular carboxyfluoroscein diacetate succinimidyl ester (CFSE; Molecular Probes, OR) and combined at a 1:1 ratio with matured DCs. Cells were washed in prefusion media and suspended in fusion media at a concentration of 2×10^7^ cells/ml. Cells were fused using the ECM 2001 pulse generator (BTX instruments, San Diego, CA). First, a low voltage alternating current of 120 V/cm for 10 seconds was applied to achieve alignment and chain formation. Then, cells were pulsed with a high voltage direct current of 1100 V/cm for 25 μs to cause reversible breakdown of cell membranes. The resulting hybrid cells were cultured overnight in CM at 37°C with 5% CO_2_. After 24 hours, the adherent cell population containing the fusion hybrids was harvested and prepared for vaccination.

### Animal studies

0.25×10^6^ D5lacZ cells/200μl of Hank’s Balanced Salt Solution were injected via tail vein into C57BL/6 mice to establish pulmonary metastases for this therapeutic model. After 2 days, 250 μg/0.5 ml HBSS of PC61 or Y13 mAb were injected into mice. One day later, animals were vaccinated with 0.3×10^6^ cells/10 μl HBSS of fusion cells that were injected into their inguinal lymph nodes. IL-12 (0.2 μg/0.5 ml HBSS) was administered intraperitoneally daily for 4 days, starting on the day of vaccination. Lungs and spleens of mice were harvested 3 weeks after establishment of pulmonary metastases. Metastases were enumerated and splenocytes were frozen for further analysis.

### ELISPOT analyses

The D5lacZ cell line, which was used as a stimulator, was cultured in the presence of 0.1 μg/ml (1,000 U/ml) murine IFN-γ for 3 days to up-regulate the expression of MHC class I and II molecules. CD4^+^ and CD8^+^ T cells were separately enriched from frozen spleen cells using their respective EasySep T cell isolation kits (Stemcell Technologies). IFN-γ cytokine secretion from stimulated T cells was detected according to the ELISPOT kit protocol provided by the manufacturer (BD Biosciences, San Jose, CA). Briefly, 96-well nitrocellulose plates were coated with purified anti-mouse IFN-γ and left overnight at 4°C. The next day, plates were washed, and blocked with CM for 2 hours. Effector cells (0.2×10^6^/well) were then co-cultured with stimulator cells at a ratio of 5:1 and incubated for 24 hours at 37°C, 5% CO_2_. After lysis of the cells, plates were developed with biotinylated detection antibody, enzyme conjugate ExtrAvidin-Alkaline phosphatase, 1/10000 dilution (Sigma-Aldrich), and substrate solution BCIP/NBT phosphatase (KPL, Gaithersburg, MD). Spots were enumerated using a CTL-ImmunoSpot analyzer (Shaker Heights, OH).

## Results

### PC61-mediated inactivation of Tregs

In a first set of experiments, the efficacy of the anti-CD25 PC61 mAb to inactivate Tregs was tested in both naïve and tumor bearing mice (Table 1). PC61 or Y13 (control) mAb was injected intraperitoneally into naive C3H/HeN mice. One mouse from each group was sacrificed at 7, 14, 21, 28, and 42 days after antibody injection. Spleens were harvested and enriched for T cells. T cells were stained with anti-CD4, anti-CD25, and anti-FoxP3 antibodies and analyzed by FACS (data not shown). Frequencies of CD4^+^CD25^+^FoxP3^+^ Tregs in both groups were compared and the results are shown in Table 1 (top). We observed that 95-98% of Tregs were undetectable for up to 21 days. After 28 days, the Treg population began to return to 50% of the original level and Treg frequencies increased to 64% of baseline after 42 days, which is conceivable given a PC61 half-life of 20 days *in vivo*.

**Table 1:** Assessment of PC61-mediated Treg inactivation

**Naive mice**		
**Day**	**Y13 (%)**	**PC61 (%)**
7	16.4	0.887
14	15.5	0.332
21	12.7	0.507
28	16.4	8.410
42	14.6	9.340
**Tumor bearing mice**		
3	7.85	1.02
10	7.40	1.60
20	11.8	1.70

*(Top)* Naïve C3H/HeN mice were injected intraperitoneally with PC61 mAb or Y13 control mAb, respectively. Mice were sacrificed at the indicated time points and splenocytes were analyzed by FACS after staining with anti-CD4, anti-CD25, and anti-FoxP3. The percentage of triple-positive cells is presented. *(Bottom)* Lung metastases were established in C57BL/6 mice via tail vein injection of D5lacZ cells. 2 days later, animals received intraperitoneal injections of PC61 or Y13 mAbs. Mice were sacrificed at the indicated time points, splenocytes were analyzed by FACS as described above and the percentage of triple-positive cells was determined.

Next, we determined the efficacy of PC61-mediated Treg inactivation in D5lacZ tumor bearing C57BL/6 mice to confirm that tumor-induced Tregs are also being inhibited by PC61 administration. D5lacZ cells were injected via tail vein into C57BL/6 mice to establish pulmonary metastases and after 2 days, PC61 or Y13 mAbs were administered intraperitoneally. Mice were sacrificed at 3, 10, 20 days after tumor inoculation and Treg inactivation was determined by FACS. As can be seen in Table 1 (bottom), 87% of Tregs were inactivated as early as 1 day after administration of PC61 mAb (day 3) and remained at that level (86%) until day 20, the typical duration of our tumor vaccination studies.

### DC-tumor cell fusion efficiency

**Figure 1:**
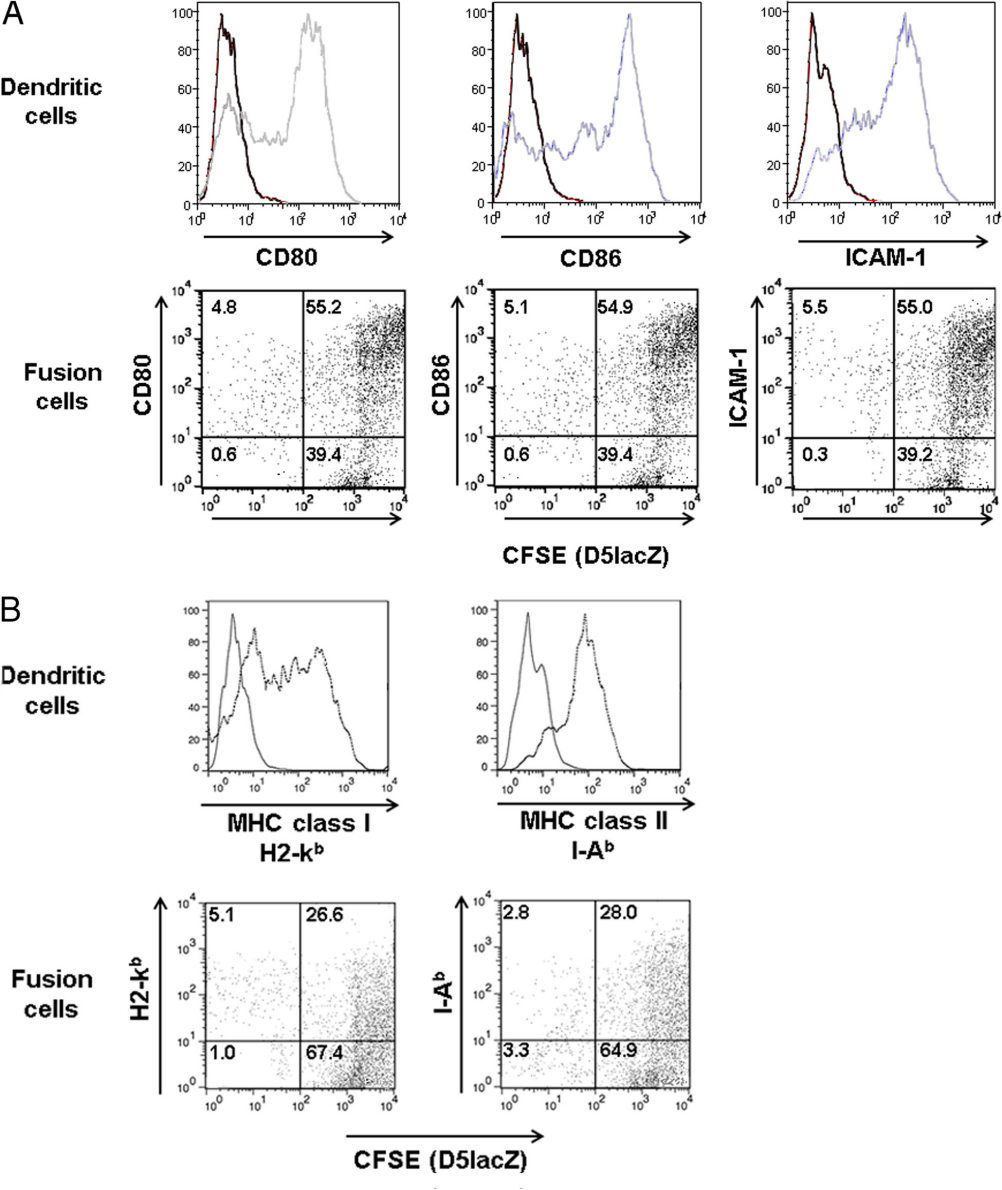
**FACS analysis of DCs and subsequent confirmation of DC-D5LacZ fusion hybrids.** (**A**) Electrofusion of DCs and D5lacZ cells. (Top panel) Phenotype of DCs at day 0 (black histograms) and day 8 (grey histograms) of culture. (Bottom panel) Prior to fusion, D5LacZ were intracellularly and covalently labeled with CFSE. 24 hours after fusion, adherent cells were harvested and stained with DC markers as indicated. Numbers in the upper right quadrant represent percentages of double-positive fusion hybrids. (**B**) MHC class I (H2-kb) and MHC class II (I-Ab) expression of DCs and DC-tumor fusion cells.

The electrofusion rate between DCs and D5lacZ melanoma cells was determined by FACS analysis. As presented in Figure 1A (top), DCs appeared to be well-differentiated and expressed high levels of maturation marker CD80, CD86 and intercellular adhesion molecule (ICAM, CD54). In contrast, cultured D5lacZ expressed none of the above-mentioned DC markers (data not shown). Prior to fusion, D5lacZ cells were intracellularly and covalently labeled with the green fluorescent dye CFSE. After electrofusion, cells were incubated in media and adherent fusion cells were harvested 24 hours later. Cells were stained with mAbs against DC markers CD80, CD86 and ICAM, for FACS analysis. Figure 1A (bottom) shows the percentages of double-positive fusion cells (upper right quadrant of dot plots). The observed fusion efficacy of about 55% was well in agreement with fusions yields routinely obtained in our laboratory. In contrast, while 78% of DCs were MHC class I positive and 84% of DCs were MHC class II positive (Figure 1B, top), only 26.6% of fusion cells expressed MHC class I molecules and only 28.0% of fusion cells were MHC class II positive (Figure 1B, bottom).

### DC-Tumor cell fusion vaccine efficacy after inactivation of Treg

**Figure 2:**
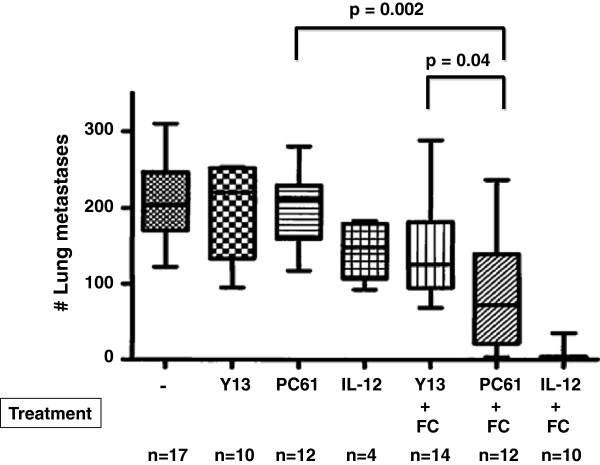
**Effects of Treg-inactivation on the efficacy of DC-tumor cell fusion vaccination.** Lung metastases were established in C57BL/6 mice via tail vein injection of D5lacZ cells. After 2 days, mice received intraperitoneal injections of saline (−), Y13 mAb (Y13), or PC61 mAb (PC61). On day 3, DC-tumor fusion cells (FC) were delivered intranodally. IL-12 was administered intraperitoneally daily for 4 days, starting on the day of vaccination. Mice were sacrificed and lung metastases were enumerated after 21 days. P-values were calculated using the two-tailed Wilcoxon rank sum test. The number (n) of animals used in each group is indicated. Results are presented as box plots in which the median, the 25 percentile, the 75 percentile, as well as minimum and maximum of each group are shown.

Next, we determined whether PC61-mediated inactivation of Tregs was capable of enhancing the efficacy of DC/tumor cell fusion vaccination in the B16 murine melanoma model (Figure 2). A subset of animals was also treated with fusion vaccine plus intraperitoneal IL-12, the current ‘gold standard’ in our murine melanoma model. Pulmonary metastases were established via tail vein injection of D5lacZ cells three days before intranodal vaccine administration and mice were sacrificed after 21 days. Mice were treated with saline (−), intraperitoneal Y13 control mAb (Y13), intraperitoneal PC61 mAb (PC61), intraperitoneal IL-12 (IL-12), Y13 mAb plus DC/D5lacZ fusion cells (Y13+FC), PC61 mAB plus DC/D5lacZ fusion cells (PC+FC), and intraperitoneal IL-12 plus DC/D5lacZ fusion cells (IL-12+FC). There was no statistically significant difference in pulmonary metastasis counts between untreated (206.4 ± 50.0), Y13- or PC61-treated animals (198.5 ± 59.17 versus 199.3 ± 49.16). However, mice treated with fusion vaccine and PC61 mAb had significantly fewer metastases (99.3 ± 89.2) than animals treated with Y13 control mAb and fusion vaccine (134 ± 74.2) (p = 0.0397) and animals treated with PC61 mAb alone (199.3 ± 49.16) (p = 0.002). We conclude that PC61 treatment synergized with fusion vaccination to increase vaccine efficacy, albeit to a lesser degree than treatment with fusion vaccine and IL-12 (4.7 ± 10.76).

### ELISPOT Analysis of IFN-γ secretion by vaccine induced CD4^+^ and CD8^+^ T cells

**Figure 3:**
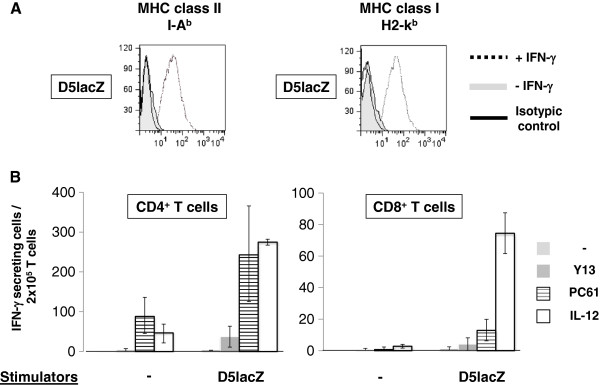
***IFN-γ ELISPOT analysis*.** (**A)** IFN-γ- stimulated D5lacZ tumor cells were used as stimulators in ELISPOT assays. Up-regulation of MHC class I (H2-k^b^) and class II (I-A^b^) was confirmed by FACS. Isotypic controls (solid lines), MHC expression in the absence of IFN-γ (filled grey histograms), and MHC expression of D5lacZ tumor cells that had been treated for 72 hours with IFN-γ at 1,000 U/ml of media (dotted lines) are presented in our FACS analyses. (**B**) Frequencies of IFN-γ-secreting CD4^+^ and CD8^+^ T-effector cells in response to D5lacZ tumor stimulation. CD4^+^ and CD8^+^ T cells were isolated from spleens that were harvested from naïve mice (naïve), mice treated with control mAb and DC/D5lacZ fusion cells (Y13+FC), mice treated with PC61 mAb and fusion cells (PC61+FC), and mice treated with systemic IL-12 and fusion cells (IL-12+FC). IFN-γ secreting cells were enumerated without stimulation (−) or after stimulation with D5lacZ cells (stimulators). 2.5×10^5^ T cells (responders) per well were stimulated at a responder to stimulator ratio of 5:1. ELISPOT assays were performed in triplicates and averaged data of 3 independent experiments are shown.

**Figure 4:**
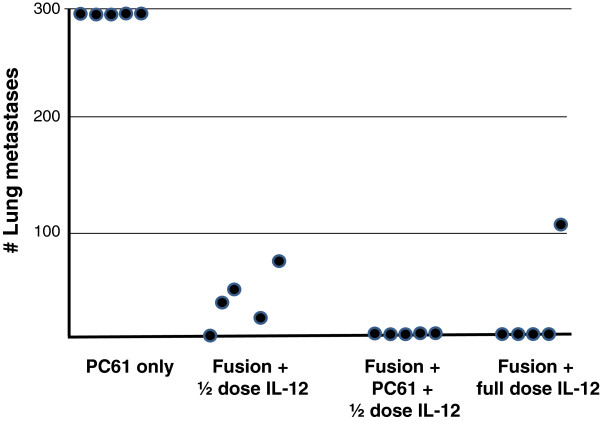
**Combination treatment with PC61 mAb and mIL-12.** Lung metastases were established in C57BL/6 mice via tail vein injection of D5lacZ cells. After 2 days, mice received intraperitoneal injections of 250 μg/0.5 ml PC61 mAb (PC61 only, Fusion + PC61 + ½ dose IL-12). On day 3, DC-tumor fusion cells (Fusion) were delivered intranodally. IL-12 was administered intraperitoneally daily for 4 days at 0.2 μg/0.5 ml (full dose) or 0.1 μg/0.5 ml (1/2 dose), respectively, starting on the day of vaccination. Mice were sacrificed after 21 days and lung metastases were enumerated.

After observing that PC61-based Treg inactivation significantly enhanced the tumor regression triggered by fusion vaccination, we sought to understand the immune mechanism by which this improvement occurred. Spleens were harvested from vaccinated animals, CD4^+^ and CD8^+^ T cells were isolated and stimulated with D5LacZ tumor cells in IFN-γ ELISPOT assays (Figure 3B). Before using D5lacZ cells as stimulators, MHC class I and class II expression was induced by treatment with IFN-γ and analyzed by FACS (Figure 3A). Data from three independent experiments were averaged, and the results reveal an overall difference in the number of vaccine-induced IFN-γ secreting CD4^+^ T cells between fusion vaccine plus Y13 mAb treatment (Y13 + FC) and fusion vaccine plus PC61 mAb treatment (PC61+FC) (37 ± 26 versus 243 ± 120) (Figure 3A). In contrast, our data indicate that there was no statistically significant difference in the number of vaccine-induced CD8^+^ T cells between the fusion + Y13 and fusion + PC61 conditions (mean=4, SD=1.54 for fusion+Y13; mean=11, SD=5.72 for fusion+PC61) (Figure 4B).

### Combination treatment with PC61 mAb and intraperitoneal mIL-12

Even though treatment with PC mAb demonstrated therapeutic efficacy against murine melanoma, it was not capable of completely eradicating pulmonary metastases. On the other hand, IL-12 administration has resulted in severe toxicity in cancer patients, hence preventing systemic application at therapeutically relevant levels. We therefore sought to determine whether PC61 mAb treatment could synergize with systemic IL-12 administration in order to decrease the dose of the cytokine needed to obtain maximal therapeutic efficacy.

Pulmonary metastases were established via tail vein injection of D5lacZ cells three days before intranodal vaccine administration with fusion cells and mice were sacrificed after 21 days. Mice were treated with intraperitoneal PC61 mAb (PC61 only), intraperitoneal IL-12 (0.1 μg) plus DC/D5lacZ fusion cells (Fusion + ½ dose), intraperitoneal IL-12 (0.1μg), plus PC61 mAB, plus DC/D5lacZ fusion cells (Fusion + PC61 + ½ dose IL-12), and with intraperitoneal IL-12 (0.2 μg) plus DC/D5LacZ fusion cells (Fusion + full dose IL-12).

As shown in Figure 4, treatment of mice with PC61 mAb alone did not lead to any reduction in tumor burden and, expectedly, administration of 0.2 μg IL-12 (full dose IL-12) in combination with fusion vaccine resulted in complete eradication of lung metastases in 4 out of 5 animals. The remaining mouse in this group appears to be an outlier. In contrast, considerable numbers of lung metastases (35.6 ± 26.1) were observed, when the administered dose of IL-12 was reduced by 50% (Fusion + 1/2 dose IL-12). Remarkably, treatment of animals with PC61 mAb, half dose IL-12 and DC/D5LacZ fusion cells led to a complete protection from lung metastasis (Fusion + PC61 + ½ dose IL-12). Thus, our data provide proof of principle that combination treatment with PC61 mAb and systemic IL-12 can indeed lower the dose of IL-12 necessary to obtain maximal therapeutic efficacy and may therefore represent a viable strategy to reduce IL-12 mediated toxicity in a clinical setting.

## Discussion

The aim of this study was to examine whether PC61 mAb-mediated functional inactivation of Tregs would enhance the potency of a DC/tumor fusion-based vaccine in a murine melanoma model with established pulmonary metastases. In agreement with previous reports, we were able to demonstrate that administration of mAb PC61 causes significant inactivation of Tregs that is maintained for about 3 weeks until the Treg population returns [20,21,25]. It is important to note that after PC61 mAb treatment only a small portion of Tregs is actually being depleted by phagocytes expressing the FcγRIII receptor [26]. The majority of Tregs is being functionally inactivated through antibody-mediated blockade of IL-2 signaling [24]. Remarkably, administration of anti-CD25 in murine models and clinical studies has not resulted in treatment related morbidity due to autoimmune disease [25], and it is tempting to speculate that tissue-residing Tregs might be affected to a lesser degree by antibody treatment than Tregs found in the lymphatic system.

Furthermore, we observed that PC61 treatment enhanced DC/tumor fusion vaccination in a synergistic fashion, and did not abrogate the induction of T-effector cells despite their transient expression of CD25 molecules. These observations are in disagreement with a recent report which examined the ability of the humanized anti-IL-2Rα mAb daclizumab to deplete T_regs_ in metastatic melanoma patients receiving antitumor vaccination with peptide- and KLH (keyhole limpet hemocyanin)-loaded DCs [27]. The authors demonstrated that while T_regs_ were effectively depleted and anti-tumor T cells were induced, daclizumab impaired the acquisition of T-effector function *in vivo*. A different report as well as our own data suggest otherwise. First, Sampson et al. [23] showed that depletion of Tregs correlated with increased vaccine-stimulated humoral immunity in glioblastoma patients during temozolomide-induced lymphopenia. Second, after mAb PC61-treatment, we observed increased frequencies of vaccine-induced CD4^+^ T cells that secreted IFN-γ upon stimulation with tumor cells. And last, administration of mAb PC61 greatly enhanced the efficacy of vaccination with fusion cells and systemic IL-12 which clearly indicates that PC61 does not seem to have any immunoinhibitory effects.

In our studies, the vaccine-induced immune response after treatment with PC61 and fusion cells consisted primarily of tumor-specific CD4^+^ T cells. This is not without precedence and in accordance with several studies, which have demonstrated that CD4^+^ T cells play a pivotal effector role during tumor rejection [28]. While the mechanisms responsible for the observed antitumor effect are still being explored, possible effector mechanisms may include Fas/FasL-mediated killing [29], interferon-γ–mediated angiostasis [30], interferon-γ–mediated restoration of the antigen-presenting pathway [31], or the recruitment or activation of innate effectors, including macrophages and eosinophils [32].

Even though, CD4^+^ T-effector cells may play an important role in anti-tumor responses, the lack of vaccine-induced CD8^+^ cytotoxic T cells in the presence of a potent Th-1-biased T-helper cell response is unsettling and may indicate that efficient cross-presentation of tumor-associated antigens did not occur in DC/tumor fusions. B16 melanoma cells are poorly immunogenic due to downregulation of transporters associated with antigen processing (TAP-1 and TAP-2) [33], resulting in reduced MHC class I expression and a diminished ability to present antigens to CD8^+^ T cells. TAP downregulation has been observed in multiple human malignancies and plays a critical role in the clinical course of malignant melanoma [34]. It is conceivable that factors leading to downregulation of TAPs in B16 melanoma cells may act in a negative trans-dominant fashion in fusion cells, thereby impairing MHC class I presentation and hence the induction of CD8^+^ T cells by DC/tumor cell hybrids. If this was true, the observed induction of CD8^+^ T-cell responses by fusion vaccine in the presence of IL-12 would be either a result of IFN-γ induced antigen-presentation by fusion cells [35] or due to stimulation of tumor-infiltrating lymphocytes and T cells in draining lymph nodes, as has been described [36,37]. Therefore, strategies aiming to overexpress TAP1 and TAP2 in fusion cells may represent a promising new approach to increase the immunogenicity of fusion vaccines.

Systemic administration of IL-12 in combination with DC-tumor fusion vaccination has demonstrated impressive anti-tumor responses in murine models. In cancer patients, however, IL-12 administration and the concomitant induction of high IFN-γ levels have resulted in severe toxicity including adverse hematopoietic, intestinal, hepatic, and pulmonary side effects, which precludes systemic application of IL-12 at therapeutically relevant levels [38]. Here, we provide proof of principle that combination treatment consisting of mAb PC61 and systemic IL-12 can lower the dose of cytokine necessary to obtain maximal therapeutic efficacy and may therefore represent a viable strategy to reduce IL-12 mediated toxicity in a clinical setting. Further studies will be needed to determine the therapeutic window of this novel approach.ken together, we conclude that PC61-mediated inactivation of Tregs is a viable strategy to improve the efficacy of DC/tumor fusion vaccination and that combining vaccination with Treg depletion and systemic administration of IL-12 may represent a novel approach to unleash the full potential of DC/tumor fusion vaccines.

## Abbreviations

CFSE: Carboxyfluoroscein diacetate succinimidyl ester; CM: Complete media; CTLA-4: Cytotoxic T-lymphocyte antigen 4; DC: Dendritic cell; ELISPOT: Enzyme-linked immunospot; FACS: Fluorescence-assisted cell sorting; FoxP3: Forkhead box transcription factor 3; GM-CSF: Granulocyte macrophage colony-stimulating factor; IFN: Interferon; IL: Interleukin: mAb, monoclonal antibody; RPMI: Roswell Park Memorial Institute; Treg: Regulatory T cell.

## Competing interests

The authors declared that they have no competing interests.

## Authors’ contributions

CT and VR equally contributed by performing experiments, data analysis, and drafting manuscript. JD and SKN made significant contributions to experimental design and manuscript revisions. JD also assisted in experiments. ED assisted in experiments and review of manuscript drafts. DST and SKP provided input on experimental design, data analysis, and manuscript revisions. WTL was responsible for experimental design, experimental supervision, data analysis, manuscript drafting and review. All authors read and approved the final manuscript.
